# Corrigendum: Microbubbles Ultrasonic Cavitation Regulates Tumor Interstitial Fluid Pressure and Enhances Sonodynamic Therapy

**DOI:** 10.3389/fonc.2022.942496

**Published:** 2022-06-17

**Authors:** Fen Xi, Yuyi Feng, Qiaoli Chen, Liping Chen, Jianhua Liu

**Affiliations:** ^1^ The First Affiliated Hospital, Jinan University, Guangzhou, China; ^2^ Department of Ultrasound Medical, Guangzhou First People’s Hospital, Guangzhou Medical University, Guangzhou, China; ^3^ The Second Affiliated Hospital, School of Medicine, South China University of Technology, Guangzhou, China

**Keywords:** ultrasound, Microbubbles, cavitation, tumor interstitial fluid pressure, SDT

In the original article, there was an error. The dosage of the sonosensitizers HMME is incorrect. 0.5mg/kg needs to be corrected to 5.0mg/kg.

A correction has been made to **Materials and Methods** *, “Microbubbles ultrasonic cavitation therapy* ***”***.

“Twenty tumor-bearing rabbits without defects related to the above contrast medium were divided into four groups (five rabbits in each group): HMME + MBUS1, HMME + US1, HMME, and blank control. Each group was treated as follows: in the HMME + MBUS1 group, each rabbit was intravenously injected with **5.0** mg/kg HMME at the ear margin 1 h later, ultrasonic emission frequency of 2.5 MPa, pulse repetition frequency of 10 Hz, a duty cycle of 0.2%, pulse emission/gap time of 9 s/3 s (The choice of this parameter is based on prior research that we are currently publishing), and irradiation time of 5 min(Shenzhen Wilde Medical Electronics Co., Ltd., models dct-700 and kht-017; effective diameter 20 mm). The probe irradiated the tumor and the SonoVue microbubbles diluted (5 mL with sterile normal saline) were slowly injected (0.5 mL/kg); in the HMME + US1 group, after each tumor-bearing rabbit was injected with the same HMME dose for 1 h, the ultrasound treatment probe was irradiated and the same volume of normal saline was slowly injected; in the HMME group, after each tumor-bearing rabbit was injected with the same HMME dose for 1 h, the ultrasound was sham irradiated for 5 min; in the blank control group, the tumor-bearing rabbits were injected with the same volume of normal saline for 1 h, then the ultrasound was sham irradiated for 5 min. The tumor IFP was measured by the WIN method before and after ultrasonic treatments**”**.

The authors apologize for this error and state that this does not change the scientific conclusions of the article in any way. The original article has been updated.

## Publisher’s Note

All claims expressed in this article are solely those of the authors and do not necessarily represent those of their affiliated organizations, or those of the publisher, the editors and the reviewers. Any product that may be evaluated in this article, or claim that may be made by its manufacturer, is not guaranteed or endorsed by the publisher.

